# Supplementary figures and images for: Effects of anesthetic management on persistent pain after breast cancer surgery
Source: PLoS One. 2025 Oct 9;20(10):e0333878. doi: 10.1371/journal.pone.0333878 (PMC12510529; doi:10.1371/journal.pone.0333878)

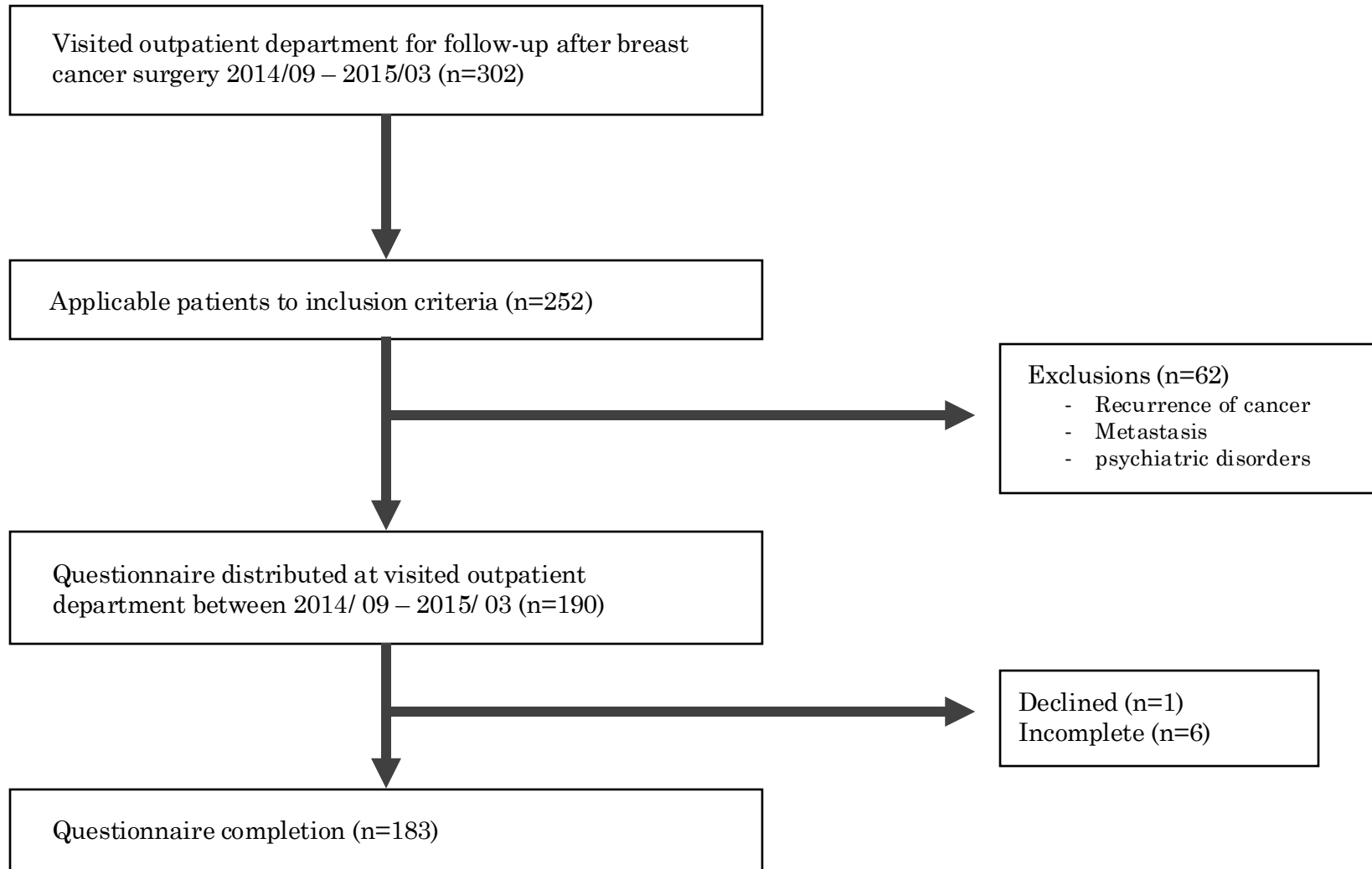

**Fig 1.** Flowchart of patient inclusion process

Supplement: S1 Fig — Detailed flowchart showing patient recruitment, inclusion/exclusion criteria application, and final study population selection from initial 302 patients to final 183 participants. (PDF) [file pone.0333878.s001.pdf]
