# Supplementary material for: Effects of anesthetic management on persistent pain after breast cancer surgery
Source: PLoS One. 2025 Oct 9;20(10):e0333878. doi: 10.1371/journal.pone.0333878 (PMC12510529; doi:10.1371/journal.pone.0333878)
Supplement: S1 File — Detailed description of all 23 variables in the S1_Dataset.csv file including variable names, definitions, coding schemes, measurement units, and value ranges for the PPBCS study dataset. (DOCX) [file pone.0333878.s003.docx]

**S1 Data Dictionary**

**Dataset**: PPBCS Study - Persistent Pain after Breast Cancer Surgery
**File**: S1_Dataset.csv
**Total observations**: 183 patients
**Total variables**: 23

**Variable Definitions**

| **Variable** | **Description** | **Values/Units** |
| --- | --- | --- |
| ID | Study participant identifier | 1-183 |
| age (yr) | Patient age at surgery | Years |
| POD (d) | Postoperative days at survey | Days |
| BMI | Body mass index | kg/m² |
| BW (kg) | Body weight | Kilograms |
| ASAPS | ASA Physical Status | 1, 2 |
| op_time(min) | Operative time | Minutes |
| Ane_time | Anesthesia time | Minutes |
| bleeding (g) | Intraoperative blood loss | Grams |
| Anes_agent | Anesthetic maintenance agent | Sevo, TIVA, Des |
| remi (μg) | Remifentanil dose rate | μg/kg/min |
| fenta (μg) | Fentanyl total dose | μg |
| ope_procedure | Surgical procedure | Bp, Bq, Bt |
| LN_procedure | Lymph node procedure | SNB, ALND |
| pecs_block | PECS block performed | No, Did |
| neoadjuvant | Neoadjuvant chemotherapy | No, Did |
| adjuvant | Adjuvant chemotherapy | No, Did |
| endocrine_therapy | Endocrine therapy | No, Did |
| radio_therapy | Radiation therapy | No, Did |
| pre_analg_use | Preoperative analgesic use | No, Did |
| pre_antidepression_antianxiety_use | Preoperative psychiatric medication | No |
| ope_analgic_use | Intraoperative analgesic use | No, Did |
| NRS | Numerical Rating Scale pain score | 0-10 |

**Code Definitions**

**Surgical Procedures (ope_procedure):**

- Bp: Partial mastectomy (breast-conserving surgery)
- Bq: Quadrantectomy
- Bt: Total mastectomy

**Lymph Node Procedures (LN_procedure):**

- SNB: Sentinel node biopsy
- ALND: Axillary lymph node dissection

**Anesthetic Agents (Anes_agent):**

- Sevo: Sevoflurane
- TIVA: Total intravenous anesthesia (propofol)
- Des: Desflurane

**Binary Variables:**

- No: Not performed/absent
- Did: Performed/present

**Notes**

- All data are de-identified
- NRS: 0 = no pain, 10 = worst pain imaginable
- Missing values: None in this dataset
- Data collection period: April 2012 - March 2014 (surgery dates)
- Survey period: September 2014 - March 2015
