# Supplementary material for: Effects of anesthetic management on persistent pain after breast cancer surgery
Source: PLoS One. 2025 Oct 9;20(10):e0333878. doi: 10.1371/journal.pone.0333878 (PMC12510529; doi:10.1371/journal.pone.0333878)
Supplement: Attachment — Submitted filename: Response to Reviewer4.docx [file pone.0333878.s005.docx]

**Response to Reviewer #4**

Dear Reviewer #4,

Thank you for reviewing our manuscript. We have carefully addressed your major concerns:

**Major Concern #1 - Study period (Line 300-302):** We fully acknowledge this significant limitation. We have enhanced our limitations section to explicitly state: "the data were collected between 2012 and 2014, a period when some current regional anesthetic techniques such as Erector Spinae Plane block (ESP) and Serratus Plane Block had not yet been formally described or incorporated into standard clinical practice." We agree this represents a serious limitation, and we have discussed how anesthetic techniques have evolved since our data collection period.

**Major Concern #2 - PECS block details (Line 282-285):** We have added comprehensive details about the PECS blocks as requested:

- Type: All blocks were PECS II blocks
- Local anesthetic: 20 ml of 0.25% ropivacaine
- Technique: Performed under ultrasound guidance with injections between the pectoralis major and minor muscles (PECS I component) and between the pectoralis minor and serratus anterior muscles (PECS II component)
- Practitioners: Performed by a limited number of anesthesiologists with expertise in regional anesthesia techniques

We have also revised our discussion to acknowledge that the limited number of patients receiving PECS block (n=9) prevents us from drawing definitive conclusions about its effect on PPBCS (Line 285-287).

We appreciate your feedback, which has helped us better acknowledge the study's limitations and provide more complete methodological details.

Sincerely,

Yu Sakai
